# The Development of Metabolomic Sampling Procedures for *Pichia pastoris*, and Baseline Metabolome Data

**DOI:** 10.1371/journal.pone.0016286

**Published:** 2011-01-21

**Authors:** Gregory D. Tredwell, Bryn Edwards-Jones, David J. Leak, Jacob G. Bundy

**Affiliations:** 1 Department of Surgery and Cancer, Imperial College London, London, United Kingdom; 2 Department of Life Sciences, Imperial College London, London, United Kingdom; Technical University of Denmark, Denmark

## Abstract

Metabolic profiling is increasingly being used to investigate a diverse range of biological questions. Due to the rapid turnover of intracellular metabolites it is important to have reliable, reproducible techniques for sampling and sample treatment. Through the use of non-targeted analytical techniques such as NMR and GC-MS we have performed a comprehensive quantitative investigation of sampling techniques for *Pichia pastoris*. It was clear that quenching metabolism using solutions based on the standard cold methanol protocol caused some metabolite losses from *P. pastoris* cells. However, these were at a low level, with the NMR results indicating metabolite increases in the quenching solution below 5% of their intracellular level for 75% of metabolites identified; while the GC-MS results suggest a slightly higher level with increases below 15% of their intracellular values. There were subtle differences between the four quenching solutions investigated but broadly, they all gave similar results. Total culture extraction of cells + broth using high cell density cultures typical of *P. pastoris* fermentations, was an efficient sampling technique for NMR analysis and provided a gold standard of intracellular metabolite levels; however, salts in the media affected the GC-MS analysis. Furthermore, there was no benefit in including an additional washing step in the quenching process, as the results were essentially identical to those obtained just by a single centrifugation step. We have identified the major high-concentration metabolites found in both the extra- and intracellular locations of *P. pastoris* cultures by NMR spectroscopy and GC-MS. This has provided us with a baseline metabolome for *P. pastoris* for future studies. The *P. pastoris* metabolome is significantly different from that of *Saccharomyces cerevisiae*, with the most notable difference being the production of high concentrations of arabitol by *P. pastoris*.

## Introduction

*Pichia pastoris* is a methylotrophic yeast commonly used for recombinant protein production. It combines the advantages of *Escherichia coli* expression systems, such as ease of use and inexpensive simple media requirements, with the ability to perform basic eukaryotic post-translational modification thus folding and processing the recombinant proteins correctly. It has recently been engineered to glycosylate proteins in a human-like manner, making the recombinant products more acceptable to the regulatory authorities [1].

Metabolomics is the systematic and comprehensive analysis of large numbers of low molecular weight compounds from a biological system. Sampling metabolites is a non-trivial problem, especially for planktonic cells, as the sampling process may perturb metabolism. Therefore, an important consideration for metabolomic investigations is the rapid “quenching” of metabolism and the following extraction of metabolites. A quenching procedure initially developed for *Saccharomyces cerevisiae* involves sampling the culture directly into cold (below −40°C) aqueous methanol (60%) followed by centrifugation to separate the intra- and extracellular metabolites [2]. A concern is the potential for solvent damage of the cell membrane, resulting in the possible leakage of intracellular metabolites. This was investigated by de Koning *et al*. through targeted (enzymatic) analyses of selected phosphorylated metabolites. They concluded that *S. cerevisiae* cells do not leak metabolites when quenched in 60% methanol at −40°C [2], and this has also been supported by several other studies [3], [4]. The situation is different for bacteria, as there have been numerous reports of metabolite leakage of intracellular metabolites from bacterial cells during the quenching process [5], [6], [7]. Bolten *et al*. reported significant leakage (>60%) of intracellular metabolites for a variety of Gram-positive and Gram-negative bacteria (*Bacillus subtilis*, *Corynebacterium glutamicum*, *Escherichia coli*, *Gluconobacter oxydans*, *Pseudomonas putida*, and *Zymomonas mobilis*) [8].

However, even for yeast, there are still contradictory reports on the efficacy of the ‘standard’ methanol quenching procedure. Villas-Boas *et al*. reported that the cell membrane of *S. cerevisiae* is damaged by contact with cold methanol, and that leakage of a number of metabolites including the organic acids phosphoenolpyruvate and *cis*-aconitate occurred; although they did confirm the earlier reports [2], [3] that metabolites including pyruvate, sugar phosphates, and nucleotides were all compatible with the quenching procedure [9]. Canelas *et al*. also reported that the standard methanol quenching method with *S. cerevisiae* resulted in metabolite leakage, and that previous studies had underestimated intracellular metabolite levels by at least twofold [10]. A recent study compared five different yeast species (*S. cerevisiae*, *Kluyveromyces marxianus*, *P. pastoris*, *Schizosaccharomyces pombe* and *Zygosaccharomyces bailii)* for metabolite leakage during the quenching protocol, but only analysed amino acids [4]. They reported that overall, quenching in cold methanol was successful, but there was significant leakage of aspartate and glutamate from *P. pastoris*.

These results highlight the need for untargeted metabolite profiling in combination with accurate quantification of metabolites in order to evaluate the validity of sampling protocols. Given this, it is surprising that there have been no evaluations of quenching and metabolite extraction that have used NMR spectroscopy. While it is true that NMR reports only on high-concentration metabolites, it has significant advantages including near-universal detection across metabolite classes, high instrument precision and highly quantitative results for inter-metabolite comparisons [11], [12], [13]. Here, we have revisited the problem of sampling and quenching for metabolome analysis in *P. pastoris*, using both ^1^H NMR and the complementary technique GC-MS, to establish efficient quenching and extraction procedures that minimise metabolite leakage. We determined absolute concentrations using NMR, giving us greater power to detect losses during the quenching procedure. Metabolite extractions with boiling ethanol or with a freeze thaw methodology with aqueous methanol are popular methods with yeast cells and these were also investigated [3], [9]. In addition, we have characterized the baseline metabolome of *P. pastoris*, by identifying the high-concentration (NMR-visible) metabolites.

## Materials and Methods

### Continuous culture

*Pichia pastoris* GS115 (*HIS4^−^*) was obtained from Invitrogen, Carlsbad, California. The continuous culture was performed in a 1.5 L fermentor (Applikon, Netherlands) at a working volume of 1.2 L. The initial glycerol batch phase was performed in basal salts medium, which was inoculated from a 30 ml overnight YPD culture grown to an OD_600_ of ∼0.7. Upon depletion of the glycerol the continuous culture was initiated with a 0.652 M methanol-containing medium at a dilution rate of 0.04 h^−1^. Steady state samples were collected after four complete volume changes at an OD_600_ of 34. The culture was supplied with 400 ml/min filtered air and the dissolved oxygen tension maintained at a minimum of 35% by variation in the impeller speed; pH was maintained at 5.0 by addition of 25% w/v potassium hydroxide; foaming was controlled with 0.01% v/v Acepol-83E (Emerald Foam Control, Hamburg).

### Media

Basal salt medium contained per litre: 26.7 ml phosphoric acid (85%), 0.93 g CaSO_4_, 18.2 g K_2_SO_4_, 14.9 g MgSO_4_.7H_2_O, 4.13 g KOH, 6 g (NH_4_)_2_SO_4_, 19.08 ml glycerol, 1 ml PTM4, 10 ml 1% w/v histidine.

Continuous culture medium [14] contained per litre 0.2 g CaCl_2_.2H_2_O, 9 ml phosphoric acid (85%), 8.5 g KOH, 6 g K_2_SO_4_, 4.67 g MgSO_4_.7H_2_O, 6 g (NH_4_)_2_SO_4_, 1 ml PTM4, and 10 ml 1% w/v histidine.

PMT4 Trace Elements Solution contained per litre 2 g CuSO_4_.5H_2_O, 0.08 g NaI, 3 g MnSO_4_.4H_2_O, 0.2 g Na_2_MoO_4_.2H_2_O, 0.02 g boric acid, 0.5 g CaSO_4_.2H_2_O, 0.5 g CoCl_2_, 7 g ZnCl_2_, 22 g FeSO_4_.7H_2_O, 0.2 g biotin, and 1 ml concentrated sulphuric acid.

### Sampling

Cell suspension (∼2 ml) from steady state continuous culture was sampled rapidly (under reduced pressure) into four different cold (<−50°C) methanol quenching solutions (13 ml): A) 60% (final conc.) aqueous methanol; B) 86% (final conc.) methanol; C) 60% (final conc.) methanol 10 mM Tricine buffer, pH 7.4; D) 60% (final conc.) methanol, 0.11 M ammonium bicarbonate. The solutions were mixed thoroughly and centrifuged for 5 min at 5000 g and −19°C (Biofuge Stratos). All samples were still below −20°C, following centrifugation. The supernatant was separated from the cell pellet and concentrated under reduced pressure. An optional cell-washing step was performed for each of the four different quenching solutions. The cell pellets were quickly vortexed in the presence of 5 ml of the appropriate cold (<−50°C) quenching solution and centrifuged as before. Intracellular metabolites were extracted from cell pellets by a cold methanol extraction procedure modified from Maharjan *et al.* [15]. Cell pellets were quickly vortexed in the presence of cold (<−50°C) 60% aqueous methanol (5 ml) and frozen in liquid nitrogen. The sample was then thawed in an ultrasonic bath for 15 min and centrifuged for 5 min at 5000 g. The supernatant was concentrated under reduced pressure and samples were stored at −80°C until analysis. In each quenching experiment, sampling was carried out threefold in rapid sequence.

### Comparison of Extraction Protocols

The efficiency of the cold methanol extraction procedure described above was compared with the boiling buffered ethanol extraction method proposed by Gonzalez *et al* [3]. Batch cultures (50 ml) of *Pichia pastoris* were grown to stationary phase at 30°C in YPD. The cultures were pooled to remove all biological variability and 6×10 ml samples were centrifuged for 5 min at 5000 g to collect the cells. Metabolites from cell pellets were extracted either by the cold methanol extraction procedure described above or by the boiling buffered ethanol extraction method. A boiling ethanol (80°C) solution containing 0.1 M tricine, pH 7 (5 ml) was added to the cell pellet and the sample incubated for 3 min at 80°C. After cooling on ice for 3 min, the solution was centrifuged for 5 min at 5000 g. The supernatant was concentrated under reduced pressure and samples were stored at −80°C until analysis. In each experiment sampling was carried out threefold in parallel.

### Biomass Estimation

We sampled cell suspensions from the fermentor under pressure, meaning that we could not exactly control the volume sampled, and so we measured the protein concentration of the cellular debris pellet after extraction in order to assess the amount of biomass sampled, following the method of Villas-Boas *et al*. [16]. Briefly, protein was solubilised from the cell pellet with the addition of 0.2 M NaOH (2 ml) and solutions were incubated at 98°C for 20 min. The solution was cooled to room temperature and protein concentration was estimated with the Bradford assay [17], relative to a BSA standard curve.

### Comparison of P. pastoris with S. cerevisiae

We compared samples from batch cultures (25 ml) of *Pichia pastoris* (NCYC 175) and *Saccharomyces cerevisiae* (NCYC 505), grown at 30°C in minimal glycerol medium (1.34% yeast nitrogen base, 1% glycerol, 4×10^−5^% biotin). The cultures were sampled during exponential growth (10 ml, two independent biological replicates), and centrifuged for 10 min at 5000 g to collect the cells. The cell pellets were extracted by the cold methanol extraction procedure described above, and analysed by NMR.

### NMR Analysis

Spectra were acquired on a Bruker Avance DRX600 NMR spectrometer (Bruker BioSpin, Rheinstetten, Germany), with ^1^H frequency of 600 MHz. Samples were introduced with an automatic sampler and spectra were acquired following the procedure described by Beckonert *et al*. [18]. Briefly, a one-dimensional NOESY sequence was used for water suppression; data were acquired into 64 K data points over a spectral width of 12 KHz, with 8 dummy scans and 512 scans per sample.

Spectra were processed in iNMR 2.6.3 (Nucleomatica, Molfetta, Italy). Fourier transform of the free-induction decay was applied with a line broadening of 0.5 Hz. Spectra were manually phased and automated first order baseline correction was applied. Metabolites were assigned using the Chenomx NMR Suite 5.1 (Chenomx, Inc., Edmonton, Alberta, Canada) relative to trimethylsilyl-2,2,3,3-tetradeuteropropionic acid (TSP). Metabolite concentrations were normalised either with respect to the protein concentration of the corresponding cellular debris pellet, or by the probabilistic quotient normalisation method described by Dieterle *et al*. [19].

### GC-MS Analysis

Samples were derivatised for GC-MS by a two-step methoximation/silylation derivatization procedure [20]. We added 2,3,3-d_3_-Leucine (20 µl, 1 mM) and U-^13^C-Glucose (20 µl, 1 mM) to the samples as derivatization standards. The dried samples were first methoximated with a solution of 40 mg/ml methoxyamine hydrochloride (10 µl) in anhydrous pyridine at 30°C for 90 min. Samples were then silylated with MSTFA (90 µl) at 37°C for 30 min. Following derivatization, 2-fluorobiphenyl in anhydrous pyridine (10 µl, 1 mM) was added to the samples as an injection standard.

GC-MS analysis was performed on an Agilent 7890 gas chromatograph connected to an Agilent 5975 MSD (Agilent Technologies UK Ltd.). Samples were injected with an Agilent 7683 autosampler injector into deactivated splitless liners according to the method of Fiehn *et al*. [20]. Metabolites were assigned using the Fiehn Library [20] with the deconvolution program AMDIS [21]. Metabolite concentrations were normalised either with respect to the protein concentration of the corresponding cellular debris pellet, or by the probabilistic quotient normalisation method described by Dieterle *et al*. [19].

## Results

### Extraction

**Figure 1:**
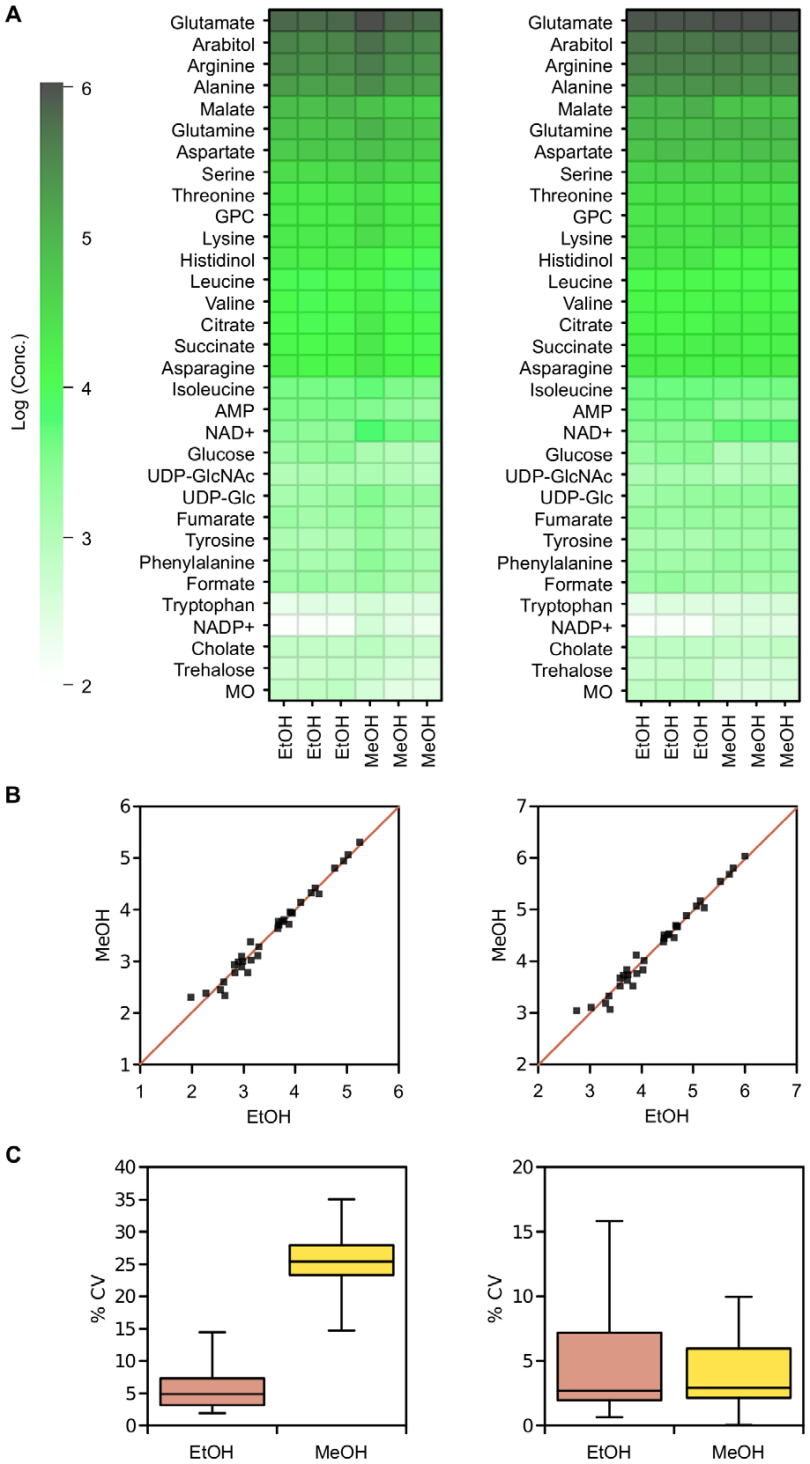
Comparison of metabolite extraction methods. A) Metabolite concentrations extracted from *P. pastoris* either with buffered boiling ethanol (EtOH) or using a freeze thaw methodology with aqueous methanol (MeOH). Concentrations were determined from ^1^H NMR spectra of the dried extracts using the Chenomx software suite. Data was normalised by either the cellular debris protein concentration (left: nM/mg cell debris protein) or by the probabilistic quotient method (right: nM) [19], and log transformed. MO  = 3-Methyl-2-oxovalerate, GPC  =  Glycero-3-phosphocholine. B) Correlation of metabolite concentrations extracted from *P. pastoris* with either buffered boiling ethanol or methanol; C) Relative standard deviation of replicate concentration measurements of metabolites extracted from *P. pastoris* with either buffered boiling ethanol or methanol.

^1^H NMR spectroscopy was used to compare metabolite extraction efficiencies from *Pichia pastoris* with buffered boiling ethanol and a freeze-thaw-ultrasonication method with aqueous methanol. The cell suspensions were sampled from the fermentor rapidly under reduced pressure, so we did not have precise volumetric measurements of samples. Therefore, it was necessary to estimate the sampled biomass by measuring the protein concentration from the cell debris pellet, which was then used to normalise metabolite concentrations. The two extraction methods had very similar extraction efficiencies, and gave essentially identical results (Figure 1A). We have throughout presented all our raw data (as heatmaps), rather than just summary statistics, to allow the reader to view the data directly. The extracted metabolites for both methods were closely correlated with each other (log_10_ concentrations), with a linear regression between them with a slope of 0.996 (R^2^ = 0.98).

While the metabolite profiles of ethanol and methanol extractions were quite similar, the coefficients of variation for metabolites from methanol extractions were relatively high (Figure 1C, left). This could have been due to additional error from the protein estimation protocol; internal normalisation, i.e. giving relative concentrations, reduced the variation between replicates to more acceptable levels (Figure 1C, right), though the metabolite concentrations were largely unchanged and the two extraction methods remained quite similar (Figure 1A, right).

### Quenching

The efficiency of current quenching procedures with *P. pastoris* was analysed by both NMR spectroscopy and GC-MS. We sampled cells from a steady-state chemostat directly into four different cold (<−50°C) aqueous methanol quenching solutions (A-D see Materials and Methods) to halt metabolism. Cells were collected by centrifugation and intracellular metabolites analysed by ^1^H NMR, or derivatised before analysis by GC-MS. For all experiments, we report metabolite concentrations normalised both with respect to the protein concentration of the cellular debris pellet, and internally (probabilistic quotient normalization, giving relative concentrations). The absolute concentrations (normalised to total protein) have the advantage that they can be easily compared across different studies, but using relative concentrations removes any additional error introduced in the protein determination, and so we have presented both sets of data.

#### Intracellular Metabolite Concentrations

**Figure 2:**
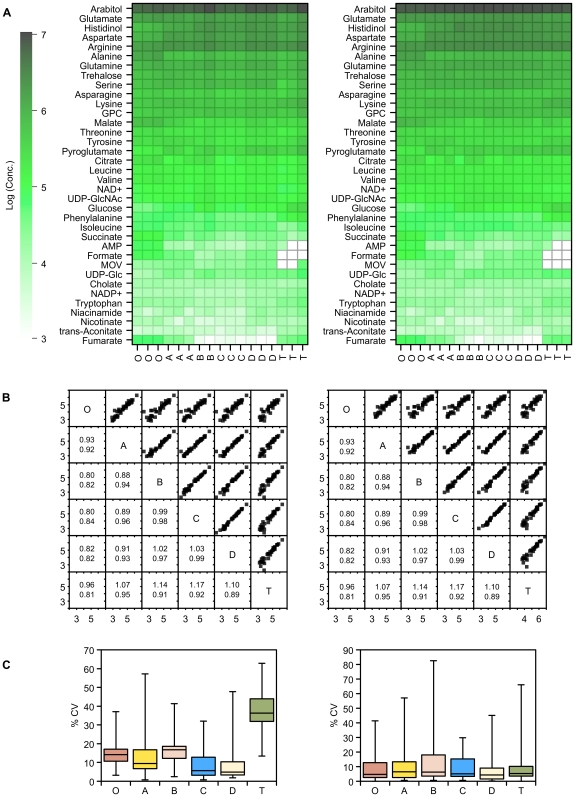
^1^H NMR measurements of intracellular metabolite concentrations following quenching. A) ^1^H NMR metabolite concentrations extracted from *P. pastoris* samples that have either been unquenched (**O**), or quenched with 60% (final conc.) aqueous methanol (**A**), 86% (final conc.) methanol (**B**), 60% (final conc.) methanol 10 mM Tricine buffer, pH 7.4 (**C**), 60% (final conc.) methanol, 0.85% ammonium bicarbonate (**D**), or quenched and extracted with the total culture (**T**). Concentrations were determined from ^1^H NMR spectra of the dried extracts using the Chenomx software suite. Data was normalised by either the cellular debris protein concentration (left: nM/mg cell debris protein) or by the probabilistic quotient method (right: nM) [19], and log transformed. MOV  = 3-Methyl-2-oxovalerate, GPC  =  Glycero-3-phosphocholine; B) Correlation matrix of metabolite concentrations (log_10_) extracted from the samples of *P. pastoris* described above. The lower half of the matrix displays the values for the gradient (above) and R^2^ (below) for the corresponding linear regression; C) Relative standard deviation of replicate measurements of metabolite concentrations extracted from the samples of *P. pastoris* cells described above.

**Figure 3:**
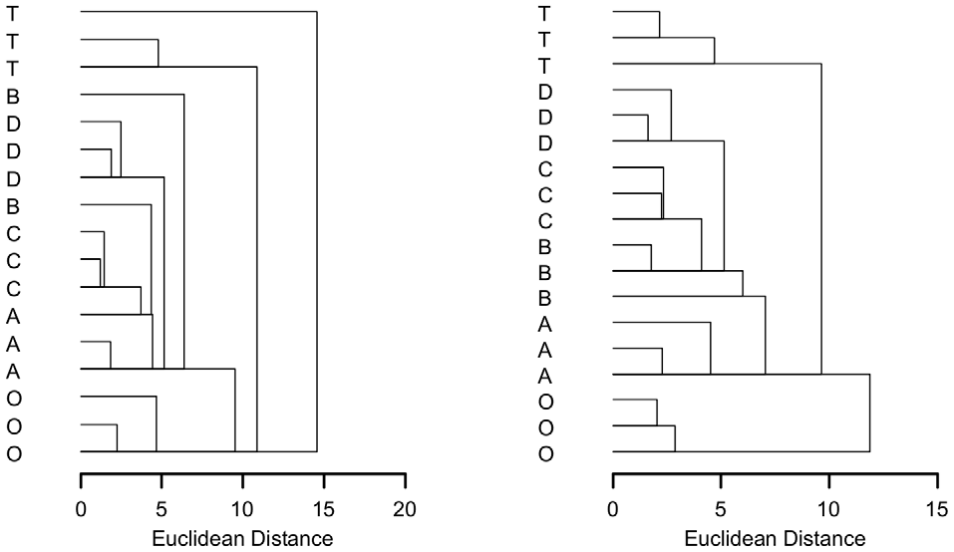
Clustering of the different quenching methods. Similarity of ^1^H NMR metabolite concentrations extracted from *P. pastoris* samples that have either been unquenched (**O**), or quenched with 60% (final conc.) aqueous methanol (**A**), 86% (final conc.) methanol (**B**), 60% (final conc.) methanol 10 mM Tricine buffer, pH 7.4 (**C**), 60% (final conc.) methanol, 0.85% ammonium bicarbonate (**D**), or quenched and extracted with the total culture (**T**); Data was normalised by either the cellular debris protein concentration (left) or by the probabilistic quotient method (right) [19].

As can be seen by inspection of the raw NMR data, either as absolute or relative concentrations (Figure 2A), there were few differences in intracellular metabolite levels between the four quenching solutions. Fumarate was found to be greatly increased in cells quenched by method A (the ‘standard’ quenching protocol). Despite this, metabolite concentrations for each of the quenching solutions correlated well with each other, with slopes for the regression line close to 1 (Figure 2B). A higher precision of replicate metabolite measurements was obtained for quenching solution D. For comparison, metabolite concentrations from unquenched (centrifuged) cells are also shown. Metabolite concentrations show an overall correlation with the quenched samples, but clearly all the quenching methods are more similar to each other than to the centrifuged samples (Figure 3). Considering specific metabolites, AMP is increased and UDP-glucose is decreased.

**Figure 4:**
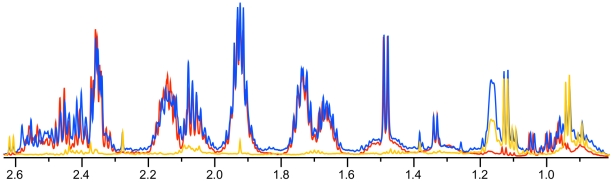
^1^H NMR spectra of extracted metabolites. Overlay of a portion of the ^1^H NMR spectra of metabolites from quenched cell extracts (red), quenched supernatants (orange), and the total quench benchmark sample (blue).

The high cell concentrations achievable in simple salts media and typical of *P. pastoris* protein production cultures^1^ gives us the option of a differential/subtractive approach for robust *in vivo* metabolite measurements. For this method we collected two samples. One sample was quenched and directly extracted into the quenching solution, i.e. without any separation of cells from medium, while the second sample was centrifuged without quenching to obtain supernatant profiles (Figure 4). By subtracting the concentrations of metabolites in the supernatant sample from the total extract, we could measure the intracellular metabolite concentrations with no possibility of losses during a centrifugation step. As a result, this total extract can serve as a benchmark, and we can determine percent loss/recovery of individual metabolites during the different quenching processes.

**Figure 5:**
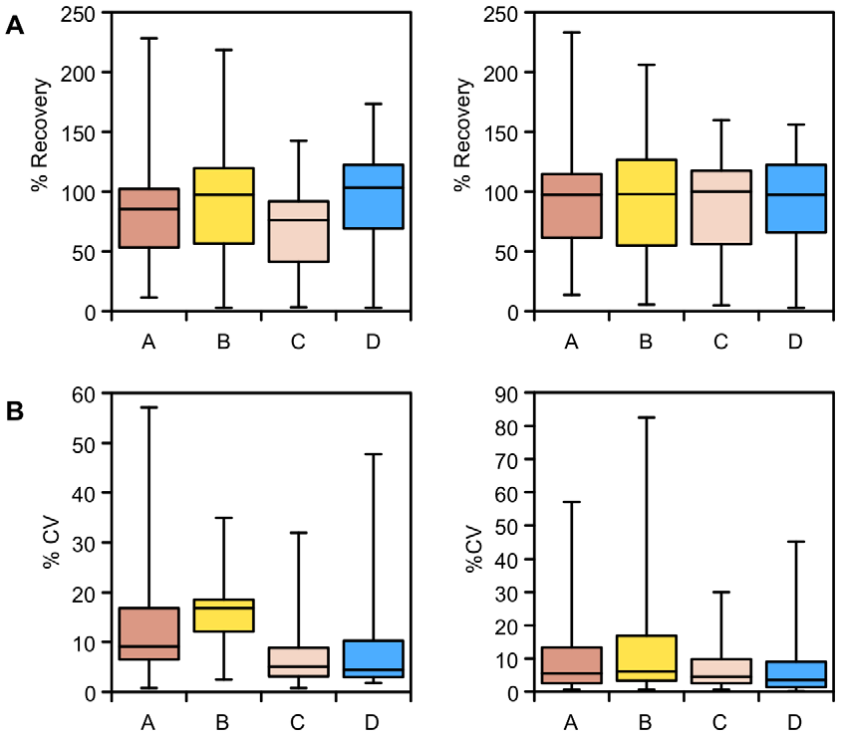
Recovery of intracellular metabolites following quenching. A) Percent recovery of intracellular metabolites extracted from *P. pastoris* samples that have either been quenched with 60% (final conc.) aqueous methanol (**A**), 86% (final conc.) methanol (**B**), 60% (final conc.) methanol 10 mM Tricine buffer, pH 7.4 (**C**), 60% (final conc.) methanol, 0.85% ammonium bicarbonate (**D**), as compared with the total culture extraction standard; B) Relative standard deviation of replicate measurements for the percent recovery of intracellular metabolites extracted from the samples of *P. pastoris* cells described above; Data was normalised by either the cellular debris protein concentration (left) or by the probabilistic quotient method (right) [19].

The metabolite levels of the total extract were very similar to those of the other quenching solutions, which included a centrifugation step (Figure 2A). Noticeable differences were that fumarate was significantly lower for quenching solutions B, C and D. However, overall, the quenching solutions B and D had metabolite recoveries distributed around 100% of the expected levels, while quenching solutions A and C showed lower recoveries (Figure 5A, left). By comparing the relative standard deviations we can see that quenching solutions C and D are more reproducible (Figure 5B, left). Normalising the data by the probabilistic quotient method reduces the differences between the different quenching solutions and improves metabolite retention for quenching solutions A and C, although quenching solutions C and D still provide lower relative standard deviations for replicate measurements. All data used for these analyses (Figures 1, 2, 3, 4 and 5) is given in supplementary information (Table S1).

**Figure 6:**
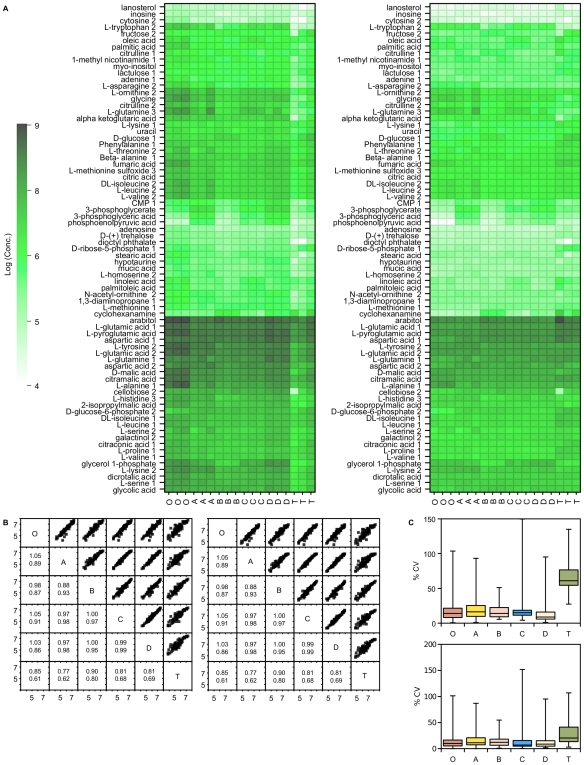
GC-MS measurements of intracellular metabolite concentrations following quenching. A) GC-MS metabolite concentrations extracted from *P. pastoris* samples that have either been unquenched (**O**), or quenched with 60% (final conc.) aqueous methanol (**A**), 86% (final conc.) methanol (**B**), 60% (final conc.) methanol 10 mM Tricine buffer, pH 7.4 (**C**), 60% (final conc.) methanol, 0.85% ammonium bicarbonate (**D**), or quenched and extracted with the total culture (**T**). Data was normalised by either the cellular debris protein concentration (left: nM/mg cell debris protein) or by the probabilistic quotient method (right: nM) [19], and log transformed; B) Correlation matrix of metabolite concentrations (log_10_) extracted from the samples of *P. pastoris* described above. The lower half of the matrix displays the values for the gradient (above) and R^2^ (below) for the corresponding linear regression. Data was normalised by either the cellular debris protein concentration (left) or by the probabilistic quotient method (right) [19]; C) Relative standard deviation of replicate measurements of metabolite concentrations extracted from the samples of *P. pastoris* cells described above. Data was normalised by either the cellular debris protein concentration (above) or by the probabilistic quotient method (below) [19].

We also analysed samples from the quenched extracts by GC-MS, and similarly to NMR the four different quenching solutions resulted in very similar metabolite concentrations overall (Figure 6A), although 3-phosphoglycerate, phosphoenol pyruvate and glycerol-1-phosphate were significantly lower in quenching solution B. Despite this however, the metabolite concentrations from different quenching solutions correlated well with each other resulting in regression lines with slopes close to 1. Interestingly, the metabolite levels for the total quench extract were significantly lower for a number of metabolites. This is likely to have been caused by effects on derivatization efficiency, leading to a large relative standard deviation for the total extract samples (Figure 6C). Quenching solution D had the smallest relative standard deviation for replicate samples.

#### Exometabolome analysis

**Figure 7:**
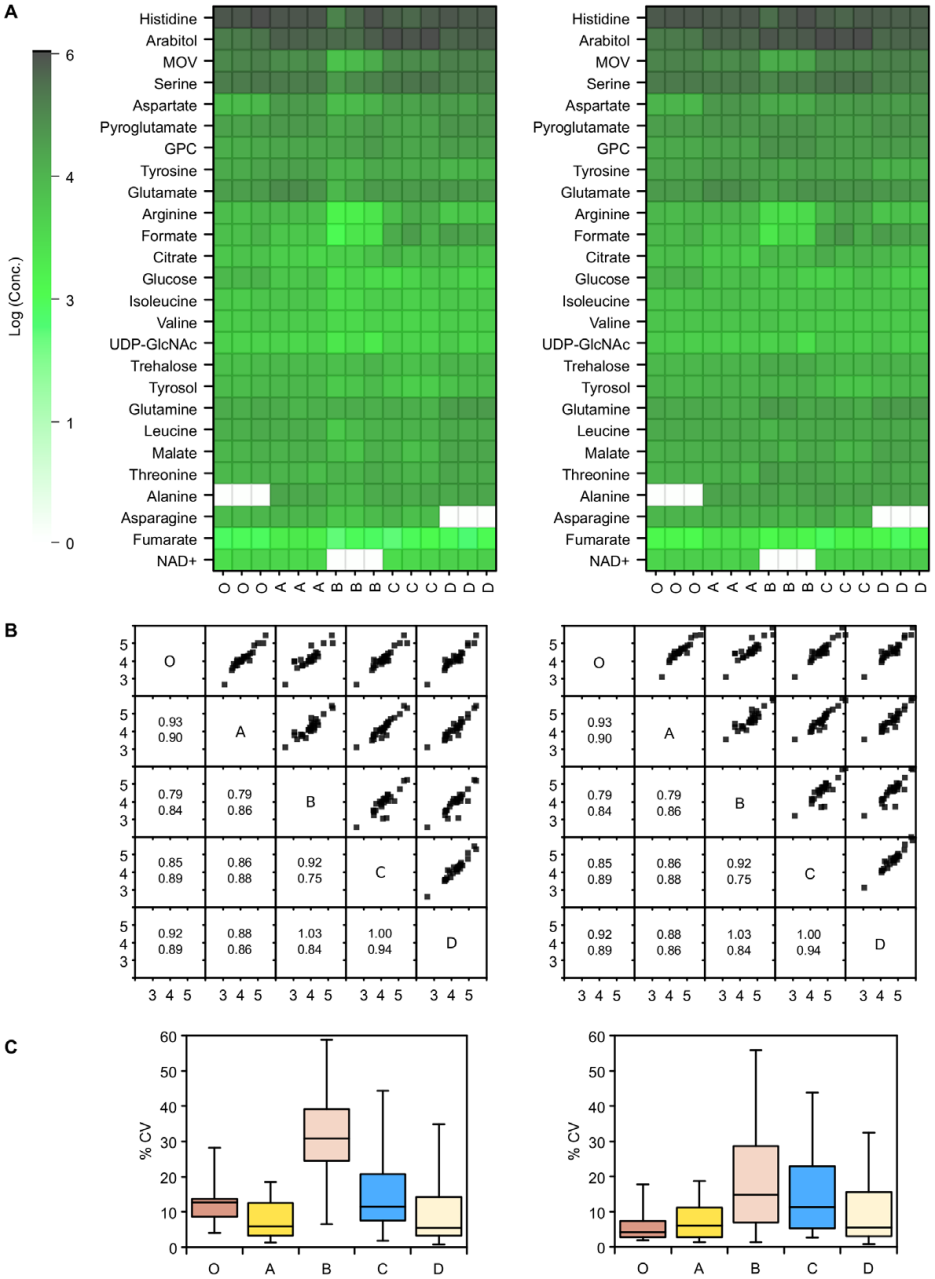
^1^H NMR measurements of extracellular metabolite concentrations following quenching. A) ^1^H NMR metabolite concentrations from supernatant samples of *P. pastoris* cultures that have either been unquenched (**O**), or quenched with 60% (final conc.) aqueous methanol (**A**), 86% (final conc.) methanol (**B**), 60% (final conc.) methanol 10 mM Tricine buffer, pH 7.4 (**C**), 60% (final conc.) methanol, 0.85% ammonium bicarbonate (**D**). Concentrations were determined from ^1^H NMR spectra of the dried extracts using the Chenomx software suite. Data was normalised by either the cellular debris protein concentration (left: nM/mg cell debris protein) or by the probabilistic quotient method (right: nM) [19], and log transformed. MOV  = 3-Methyl-2-oxovalerate, GPC  =  Glycero-3-phosphocholine; B) Correlation matrix of metabolite concentrations (log_10_) extracted from the various samples of *P. pastoris*. The lower half of the matrix displays the values for the gradient (above) and R^2^ (below) for the corresponding linear regression; C) Relative standard deviation of replicate measurements of metabolite concentrations extracted from the supernatant samples of *P. pastoris* cultures described above.

We measured extracellular (supernatant) metabolite concentrations to make it easier to identify metabolites that leaked during the quenching process. The metabolite levels in the supernatants of each of the quenching solutions generally compare well with the unquenched supernatant standard (Figure 7). However, a few metabolites are significantly different. Alanine was not found in the unquenched sample but appeared in the supernatants of all quenching solutions, and arabitol was higher in quenched supernatants for all solutions. Aspartate was also significantly higher in quenching solutions A, C and D. NAD was not present in the supernatants of solution B, which may perhaps be due to a reduced recovery. We could not confidently assign asparagine in the ^1^H NMR spectra of the quenched supernatant sample from solution D due to overlapping peaks. Interestingly, using quenching solution A gave higher intra- and extracellular concentrations of fumarate. Despite these differences in some individual metabolites, overall the profiles of the different quenching solutions correlated well with each other, with regression lines with slopes close to 1. The reproducibility was slightly better for quenching solutions A and D, as shown by the distributions of relative standard deviations.

**Figure 8:**
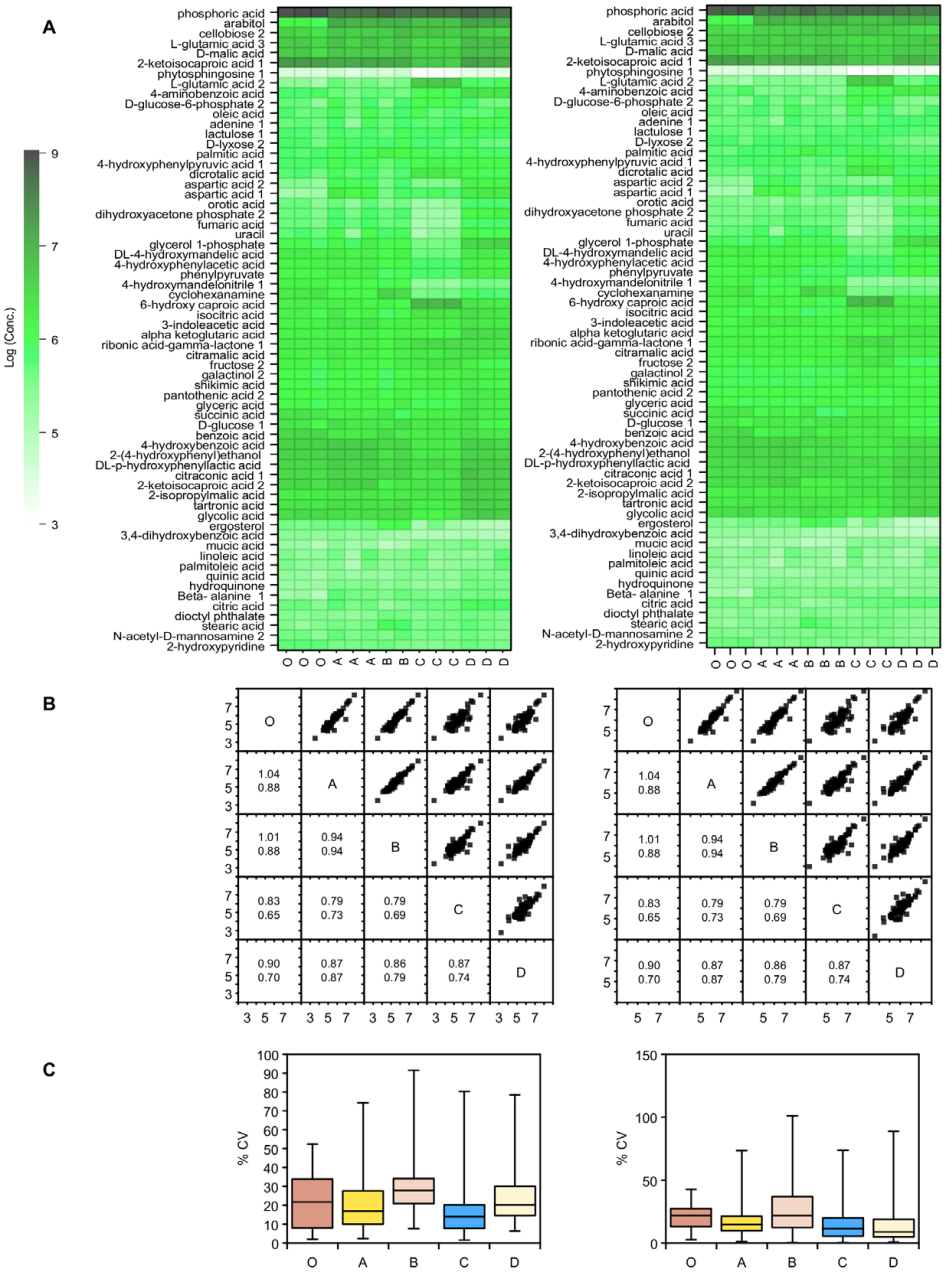
GC-MS measurements of extracellular metabolite concentrations following quenching. A) GC-MS metabolite concentrations from supernatant samples of *P. pastoris* cultures that have either been unquenched (**O**), or quenched with 60% (final conc.) aqueous methanol (**A**), 86% (final conc.) methanol (**B**), 60% (final conc.) methanol 10 mM Tricine buffer, pH 7.4 (**C**), 60% (final conc.) methanol, 0.85% ammonium bicarbonate (**D**). Data was normalised by either the cellular debris protein concentration (left: nM/mg cell debris protein) or by the probabilistic quotient method (right: nM) [19], and log transformed; B) Correlation matrix of metabolite concentrations (log_10_) extracted from the various samples of *P. pastoris*. The lower half of the matrix displays the values for the gradient (above) and R^2^ (below) for the corresponding linear regression; C) Relative standard deviation of replicate measurements of metabolite concentrations extracted from the supernatant samples of *P. pastoris* cultures described above.

In addition to the NMR-based profiling, we used GC-MS as a complementary analytical technique for the exometabolome data (Figure 8). The metabolite profile from quenching solution C was quite different from the other quenching solutions, giving significantly lower concentrations for a number of metabolites. This is presumably the result of interference by the tricine buffer, e.g. in the derivatization process. However, the levels of metabolites in the other quenching solutions correlated well with the unquenched supernatant sample as well as with each other. Arabitol was higher in all quenched supernatants for all solutions, consistent with the NMR results. Glutamate and aspartate concentrations were also found to be higher for quenched supernatants compared with the unquenched supernatant standard.

**Figure 9:**
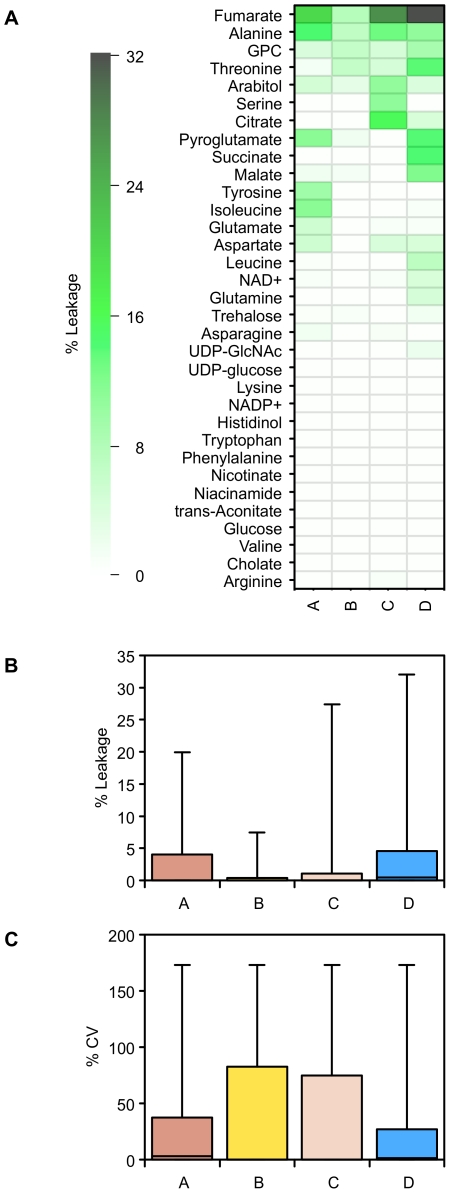
^1^H NMR measurements of metabolite leakage following quenching. A) ^1^H NMR data for the percent leakage of intracellular metabolites extracted from supernatant samples of *P. pastoris* cultures that have either been quenched with 60% (final conc.) aqueous methanol (**A**), 86% (final conc.) methanol (**B**), 60% (final conc.) methanol 10 mM Tricine buffer, pH 7.4 (**C**), 60% (final conc.) methanol, 0.85% ammonium bicarbonate (**D**), as compared with the unquenched supernatant standard; B) Distributions of the percent leakage of intracellular metabolites for the samples described above; C) Relative standard deviation of replicate measurements for the percent leakage of intracellular metabolites extracted from the samples of *P. pastoris* supernatants described above. GPC  =  Glycero-3-phosphocholine.

**Figure 10:**
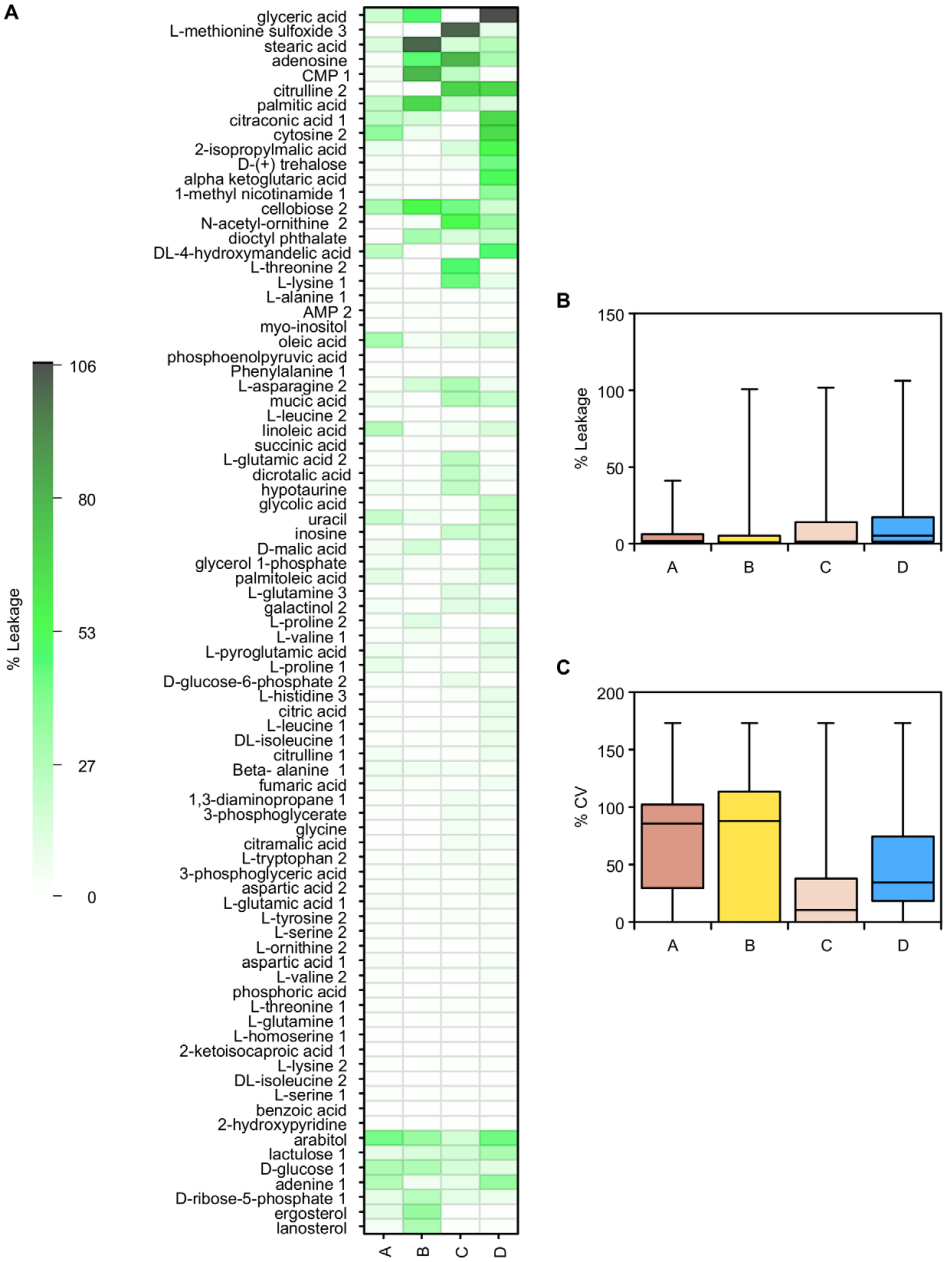
GC-MS measurements of metabolite leakage following quenching. A) GC-MS data for the percent leakage of intracellular metabolites extracted from supernatant samples of *P. pastoris* cultures that have either been quenched with 60% (final conc.) aqueous methanol (**A**), 86% (final conc.) methanol (**B**), 60% (final conc.) methanol 10 mM Tricine buffer, pH 7.4 (**C**), 60% (final conc.) methanol, 0.85% ammonium bicarbonate (**D**), as compared with the unquenched supernatant standard; B) Distributions of the percent leakage of intracellular metabolites for the samples described above; C) Relative standard deviation of replicate measurements for the percent leakage of intracellular metabolites extracted from the samples of *P. pastoris* supernatants described above.

The supernatant data can be used to estimate metabolite leakage during the quenching process, expressed as a percentage of the unquenched supernatant concentrations (Figures 9, 10). The NMR data show that overall solutions B and C had a much smaller level of metabolite leakage than the other quenching solutions. Even so, for quenching solutions A and D, the losses were generally small: 75% of the metabolites that leaked were below 5% of their intracellular levels. Despite this, however, the results using quenching solutions A and D gave a much smaller relative standard deviation for replicate samples. Fumarate was the main metabolite with a high percent leakage; the other metabolites were below 15%.

The GC-MS data gave a similar picture (Figure 10). Although a few metabolites leaked into the quenching solutions at relatively high levels, the majority were at low levels compared to their intracellular concentration. Quenching solutions A and B overall had the lowest levels of intracellular metabolite leakage, but even for C and D, 75% of metabolites found in the quenching supernatant were at less than 15% of their intracellular levels.

#### Wash Step

**Figure 11:**
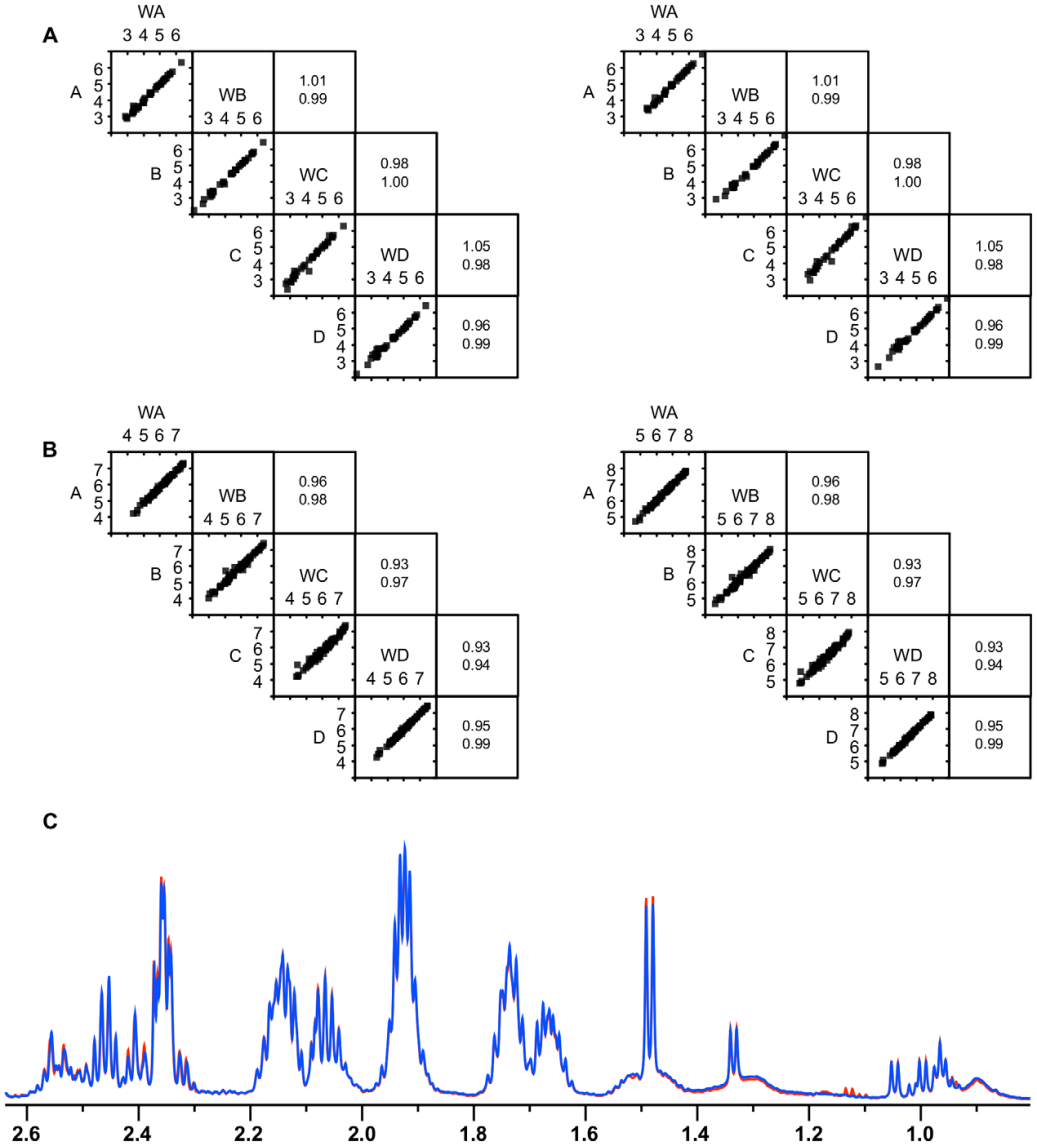
Evaluation of the wash step following quenching. A) Correlations of ^1^H NMR metabolite concentrations for the washed quenched extracts (WA, WB, WC and WD), with their corresponding unwashed quenched extracts from *P. pastoris* cultures that have either been quenched with 60% (final conc.) aqueous methanol (**A**), 86% (final conc.) methanol (**B**), 60% (final conc.) methanol 10 mM Tricine buffer, pH 7.4 (**C**), 60% (final conc.) methanol, 0.85% ammonium bicarbonate (**D**). Data was normalised by either the cellular debris protein concentration (left: nM/mg cell debris protein) or by the probabilistic quotient method (right: nM) [19], and log transformed. The squares on the right display the values for the gradient (above) and R^2^ (below) for the corresponding linear regression; B) Correlations of GC-MS metabolite concentrations for the washed quenched extracts with their corresponding unwashed quenched extracts from *P. pastoris* cultures. Data was normalised by either the cellular debris protein concentration (left: nM/mg cell debris protein) or by the probabilistic quotient method (right: nM) [19], and log transformed; C) Overlay of a portion of the ^1^H NMR spectra of metabolites from quenched cell extracts (red) and quenched cells that have been through a wash step before extraction (blue).

We investigated the usefulness of an additional wash step following quenching with the four different quenching solutions. Correlations of the washed quenched cell extracts with the quenched cell extracts had slopes close to 1, indicating that no significant leakage occurred during the wash step (Figure 11).

Direct visual comparison of spectra for washed and unwashed quenched cell extracts shows that the profiles are extremely similar (Figure 11C). Notably, the unwashed quenched cells did not contain a significant amount of resonances from contaminating extracellular metabolites – although washing the cells reduced a small number of signals between 1.08 and 1.2 ppm, including resonances from 3-methyl-2-oxovalerate, this spectral region does not contain a lot of signals from other intracellular metabolites.

#### Baseline Metabolome of *Pichia pastoris*

In this study we have thoroughly characterised the high concentration metabolites in both the extra- and intracellular locations by NMR spectroscopy (Figure 2A). The assignments of metabolites were confirmed through the use of various 2D NMR techniques such as COSY, TOCSY and HSQC and spike in experiments. In addition to this we also used GC-MS as a complementary analytical tool to extend the detection to lower concentrated metabolites (Figure 6A). This has provided us with a baseline metabolome for *P. pastoris* for future studies.

In comparison with *S. cerevisiae*, intracellular metabolite profiles of exponentially growing *P. pastoris* are clearly different. The NMR profiles of the two yeast species grown on the same medium are clearly different (Figure S1), although it should be noted that these spectra were obtained from centrifuged rather than quenched cells. One of the most obvious metabolic differences is that *P. pastoris* cells produce arabitol, a five-carbon sugar alcohol, whereas *S. cerevisiae* cells do not. This metabolite, which has the highest intracellular concentration in *P. pastoris*, has been identified in other yeast species, such as *Pichia anomala* [22] and *Pichia sorvitophila* [23], and may be involved in regulating osmotic conditions within the cell [23]. This job may also be shared by trehalose, which is the next highest concentrated carbohydrate within *P*. *pastoris* cells and is also present in *S. cerevisiae*. This metabolite is believed to be responsible for controlling osmotic conditions in *S. cerevisiae* and in a variety of other yeasts and bacteria [23], [24], and contributes to survival during various stress conditions such as heat, freezing, dehydration and desiccation by acting as a membrane protectant [24]. We have also confirmed the assignment of a number of nucleotide sugars in *P. pastoris*, such as UDP-glucose and UDP-*N*-acetylglucosamine. As the strains we are currently working with are histidine auxotrophs, a relatively high concentration metabolite within *P. pastoris* cells is histidinol. Such mutants lack histidine dehydrogenase (HIS4) and are unable to convert histidinol into histidine, the last step in its biosynthesis. A interesting extracellular metabolite that has been identified in *P. pastoris* cultures is tyrosol [2-(4-hydroxyphenyl)ethanol]. Tyrosol, which is derived from tyrosine, is a known quorum-sensing molecule in *Candida albicans* that stimulates the formation of germ tubes [25]. There is a growing recognition of the potential importance of quorum sensing in yeasts, although to date the only evidence within *Pichia* species is that the induction of pyruvate decarboxylase is density-dependent for *P. stipitis* (although a signalling molecule was not identified) [26].

In an attempt to evaluate the equivalency of the two analytical platforms, we calculated correlations between NMR and GC-MS concentrations for individual metabolites (supplementary information, Figure S2). While not all metabolites were highly correlated across the two techniques, it must be noted that all the samples were obtained under very similar biological conditions from a steady-state chemostat fermentor. As such the samples should contain very little biological variation and therefore, correlations would obviously be lower than for studies where there was a lot of metabolite variation between studies. Previous studies with NMR and GC-MS however have shown a high degree of comparability between the two platforms for metabolites that are detected by both methods [27].

## Discussion

The vast diversity of cellular structures and intra- and extracellular metabolites necessitates the validation of metabolomic sampling protocols for different organisms. While yeast species such as *S. cerevisiae* have been well studied, only one previous paper has investigated sampling conditions with *P. pastoris* [4]. However, this was a targeted analysis of amino acids, and much confusion in the literature about the validity of quenching protocols has resulted from such targeted analyses. Here, we used both ^1^H NMR and GC-MS as untargeted profiling tools to evaluate extraction and quenching protocols with *P. pastoris*. We chose NMR because it has high analytical precision for a wide range of metabolites, and can readily be used to determine absolute concentrations, and so is particularly well suited to comparing sample preparation methods which may result in only subtle metabolic differences. Because NMR is limited to reporting on only a small set of high-concentration metabolites, we also used GC-MS as a complementary tool to extend metabolome coverage.

For metabolomic investigations, it is vital to get accurate measurements of *in vivo* metabolites representing all functional categories, and therefore efficient extraction methodologies are necessary. Our results indicate that extraction with buffered boiling ethanol is just as efficient as the freeze-thaw methodology with aqueous methanol plus sonication. Given this, we preferred the freeze-thaw aqueous methanol extraction for a number of reasons. The protocol is simpler and does not require heating, which therefore prevents the potential degradation of heat-labile compounds. The boiling ethanol extraction method also required an additional step as we observed an insoluble precipitate following resolubilization with the NMR buffer, and filtering was required to remove it. Furthermore, tricine is used as a buffer in the boiling ethanol extraction, which results in peaks at 3.67 and 3.76 ppm in the NMR spectra of cell extracts.

The cell suspensions were sampled rapidly from the fermentor under pressure directly into chilled solvent. As a result, we could not sample precise volumes, and so needed to normalise the data to allow for this. One method was by estimating the amount of biomass that was sampled by measuring the total protein content of the cellular debris pellet following metabolite extraction. This has the great advantage that it gives metabolite concentrations in a form that are directly comparable across different studies; however, it also means that the process of measuring the protein concentration is also a potential source of error. This is exemplified by Figure 1, where one of the methanol extract replicates showed consistently higher concentrations for all metabolites extracted, resulting in a much higher relative standard deviation compared with the ethanol extract replicates. Because of this, we also normalized all the data internally, i.e. expressing all data as relative concentrations. This removes any variation introduced by the protein determination, and hence provides the best comparison between sampling protocols for metabolome analysis. For completeness both normalisation methods have been applied to all data for comparison.

The high turnover of intracellular metabolites highlights the need for reliable, reproducible quenching techniques for microbial metabolomics. A number of methods have been proposed to address the problem of metabolite leakage during quenching. Canelas *et al*. reported that higher concentrations of methanol might reduce metabolite losses, rather than increase them [10]. Several methods attempt to reduce osmotic shock through the addition of salts and buffers such as tricine, HEPES and PIPES [28], [29]. However, many of these may cause undesirable ion suppression effects in mass spectrometry studies, or else lead to unwanted resonances in NMR spectra. For mammalian cells, 60% methanol with ammonium bicarbonate was found to result in the greatest recovery of the metabolites being measured [29], although a recent paper reported contradictory results [30].

Unfortunately, it is not a trivial matter to evaluate different quenching techniques, and this has led to confusion in the literature as to the effectiveness of quenching protocols. Some studies have tested expected metabolite ratios, such as measuring the adenylate energy charge. However, although this could identify cases where *in vivo* metabolite ratios had been disrupted, it could not by itself confirm the success of quenching protocols (e.g. there could be metabolic changes that would still maintain the AEC). Furthermore, this focuses on highly labile metabolites, but says nothing about possible changes in the rest of the metabolome. Alternatively, we could compare intracellular levels of other metabolites to see if there were any significant losses, but the *in vivo* value would not be known ahead of time. Conversely, metabolite concentrations in supernatants of quenched samples could be compared, with any significant increases indicating metabolite leakage. However, if a metabolite leaks for all quenching protocols being compared, then this would go unnoticed. Therefore, it would be more convenient to compare quenching methods to a “gold standard” of intracellular metabolite concentrations, which unfortunately does not exist. A close approximation would involve direct quenching and extraction of the culture broth (i.e. cells + supernatants, therefore removing the problematic washing step) and a differential/subtractive method for estimation of intracellular metabolite concentrations [31]. However, this method may not be suitable for all microbial cultures. Given that the cytosolic volume is much smaller than the total culture volume for most microbial experiments, this can lead to very small intracellular metabolite peaks of interest against very high background peaks from the growth medium. *P. pastoris* can be grown at extremely high cell densities in a simple salts medium, and therefore offers the great advantage that ‘total’ quenching of the cells + broth combined can give a good picture of the endometabolome, even when using an untargeted method such as NMR (Figure 4). The supernatant sample contains only very low concentrations of metabolites compared with the quenched cell extract sample, and luckily resonances from extracellular metabolites, for example signals at 1.17, 1.38, 2.28 and 2.61 ppm, do not overlap greatly with intracellular metabolite resonances. In other words, total quenching offers us a gold standard method to compare against for *P. pastoris*.

There were subtle differences between the four quenching solutions, e.g. metabolites such as fumarate fell well below their estimated intracellular concentrations for methanol quenching, but a broad comparison showed that, overall, they all gave very similar results. The intracellular metabolite concentrations from quenched extracts also correlated well with the total quenching ‘gold standard’. While the total quenching method worked well for ^1^H NMR, it was not as effective for GC-MS. Many metabolites had apparently lower concentration than in the quenched extracts, which was presumably the result of the high amounts of salts as well as metabolites interfering with the derivatization process.

We could get an accurate estimation of extracellular metabolites, through centrifugation of an unquenched culture sample. This simplicity is part of the advantage of metabolic footprinting [32], and gives a reliable standard for comparison of the exometabolome data from the quenched samples. This showed that extracellular alanine and arabitol both increased following the quenching protocol, indicating leakage from cells. This is consistent with other studies; Bolten *et al*. [4] reported leakage of aspartate and glutamate from *P. pastoris* following the standard methanol quenching procedure. Our ^1^H NMR results confirm the increase of aspartate in the 60% methanol (A), 60% methanol, 10 mM tricine (C) and 60% methanol, 0.11 M ammonium bicarbonate (D) quenching solutions, though glutamate was not significantly increased. However, the GC-MS results showed significantly increased levels of both aspartate and glutamate in the quenching solutions.

Given the evidence for metabolite leakage during quenching, it is useful to represent the increased extracellular metabolite concentrations relative to their intracellular concentration. The ^1^H NMR results suggest that the leakage of metabolites was at a low level, with increases in the quenching solution below 5% of their intracellular level for 75% of metabolites. The GC-MS results suggest a slightly higher level with increases below 15% of their intracellular levels.

Current quenching protocols include a wash step to ensure the complete removal of the extracellular metabolites. However, this extra step may risk further metabolite leakage or may result in metabolite turnover. With the current conditions (high cell concentrations, salts medium) the wash step did not seem to provide a significant advantage to warrant its inclusion. While, no further significant leakage occurred during the washing procedure, the unwashed quenched cell extracts did not contain large concentrations of contaminating extracellular metabolites. Furthermore, in the ^1^H NMR spectra these metabolites did not greatly overlap with intracellular metabolite signals (Figure 11C).

From these results it is difficult to recommend one quenching solution over another, as each of the methods have resulted in similar intra- and extracellular profiles. Parsons *et al*. have proposed using median relative standard deviation as a practical benchmark [33]. In this case the 60% methanol, 0.11 M ammonium bicarbonate (D) quenching solution showed lower relative standard deviation values for both intra- and extracellular metabolite levels. Therefore, this method perhaps provides an improvement over the other quenching methods. For mass spectrometry studies, further consideration should be given to any buffer additives to the quenching solution. The GC-MS analysis of extracellular metabolites from the 60% methanol, 10 mM tricine (C) quenching solution, resulted in low concentrations for a number of metabolites compared with the other quenching solutions. This was perhaps a result of interference of the tricine buffer, e.g. in the derivatization process, though surprisingly this was not evident for quenching solution D, which contained ammonium bicarbonate.

In summary, we have performed a comprehensive investigation of appropriate sampling techniques for *P. pastoris* using NMR and GC-MS, which led to an untargeted and highly quantitative analysis of intra- and extracellular metabolites. We have identified the major high-concentration metabolites found in *P. pastoris* during exponential growth. Quenching protocols based on the common procedure using cold methanol did cause some metabolite losses from cells, but the losses were fairly small compared to the remaining intracellular concentrations, and so the technique can still be useful if small losses can be tolerated. Total culture extraction was an efficient sampling technique for *P. pastoris* for NMR analysis, but salts in the media affected the GC-MS analysis. There was no benefit to including an additional washing step in the quenching process, as the results were essentially identical to those obtained just by a single centrifugation step.

## Supporting Information

Figure S1 **Metabolite differences between** ***Saccharomyces cerevisiae*** **and** ***Pichia pastoris*** **.** 600 MHz ^1^H NMR spectra of cell extracts of *S. cerevisiae* (blue) and *P. pastoris* (red). Two independent biological replicates of each are shown. Specific metabolites highlighted include alanine, glutamine and betaine (higher in *P. pastoris*), arabitol (not detected in *S. cerevisiae*) and leucine, threonine and phenylalanine (higher in *S. cerevisiae*).
(TIF)

Figure S2 **Comparison between NMR and GC-MS for metabolite profiling**. Correlation (*R* ^2^) between the two techniques for metabolites detected by both methods. Blue line indicates statistically significant relationship (*P* = 0.05).
(TIF)

Table S1 **Baseline metabolome data for** ***Pichia pastoris*** **, as detected by ^1^H NMR**. Column 1 (blue shading) shows average values as % of highest concentration metabolite (arabitol) for data obtained by quenching in 60% methanol +0.85% NH_4_HCO_3_, in order to give a quick comparison. Remaining columns indicate fitted metabolite data used for all statistical analyses. No shading  =  concentration in nM/mg protein; grey shading  =  relative concentrations, data normalized by probabilistic quotient method.
(XLSX)
