# Supplementary figures and images for: The Development of Metabolomic Sampling Procedures for Pichia pastoris, and Baseline Metabolome Data
Source: PLoS One. 2011 Jan 21;6(1):e16286. doi: 10.1371/journal.pone.0016286 (PMC3025026; doi:10.1371/journal.pone.0016286)

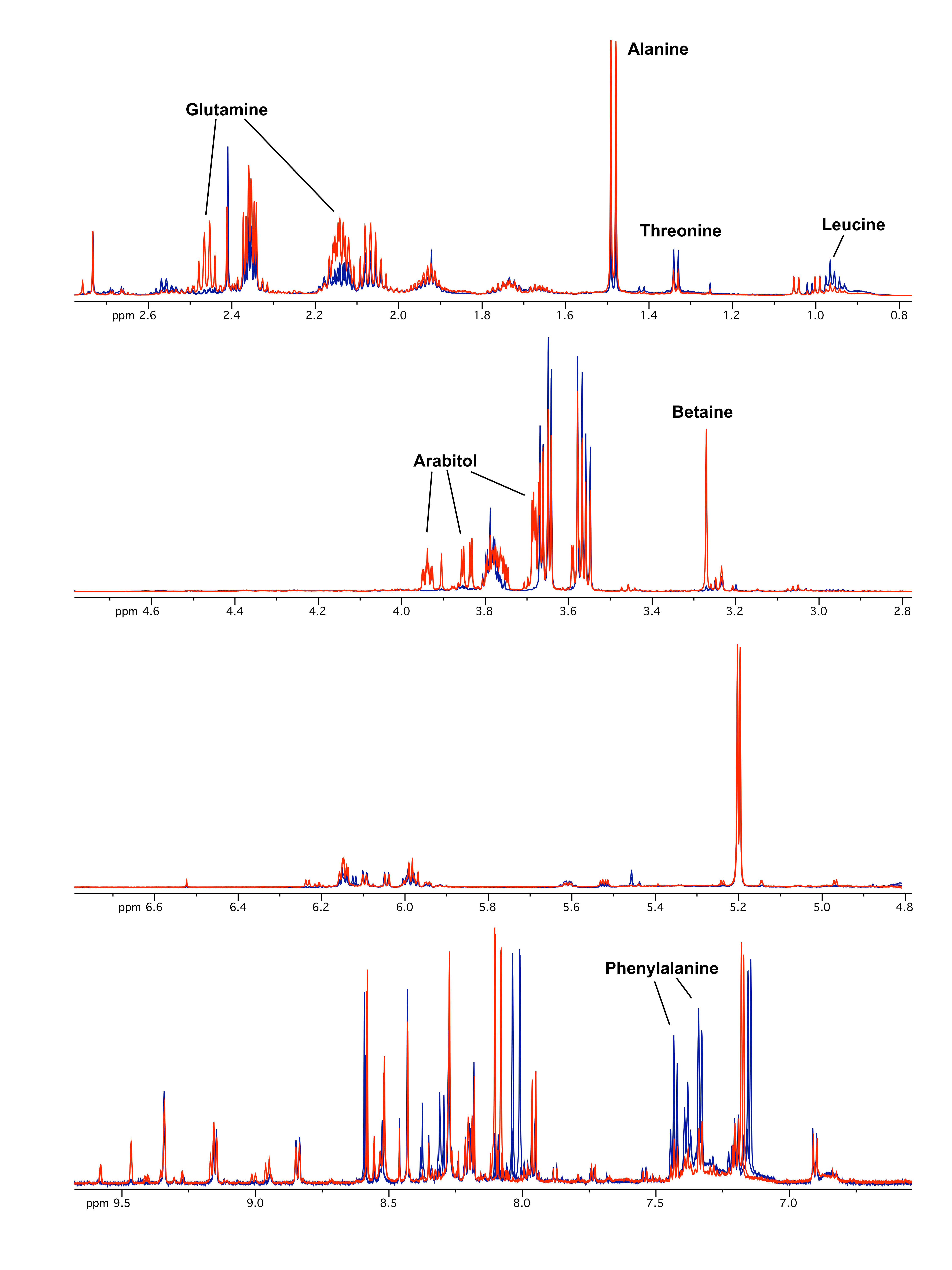

Supplement: Figure S1 — Metabolite differences between Saccharomyces cerevisiae and Pichia pastoris . 600 MHz 1H NMR spectra of cell extracts of S. cerevisiae (blue) and P. pastoris (red). Two independent biological replicates of each are shown. Specific metabolites highlighted include alanine, glutamine and betaine (higher in P. pastoris), arabitol (not detected in S. cerevisiae) and leucine, threonine and phenylalanine (higher in S. cerevisiae). (TIF) [file pone.0016286.s001.tif]

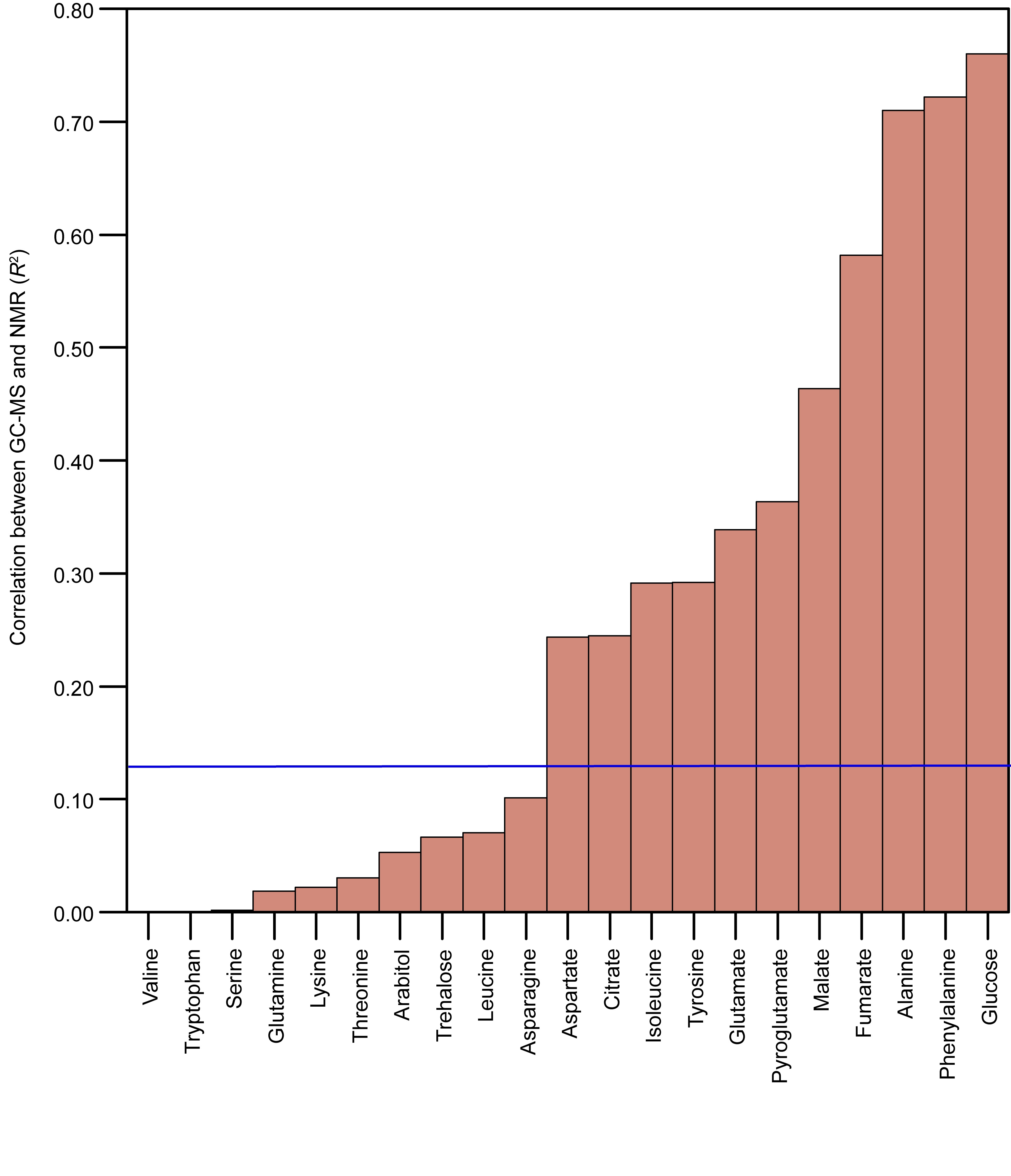

Supplement: Figure S2 — Comparison between NMR and GC-MS for metabolite profiling. Correlation (R 2) between the two techniques for metabolites detected by both methods. Blue line indicates statistically significant relationship (P = 0.05). (TIF) [file pone.0016286.s002.tif]
